# Effects of TiO_2_ Nanoparticles on the Corrosion Protection Ability of Polymeric Primer Coating System

**DOI:** 10.3390/polym13040614

**Published:** 2021-02-18

**Authors:** Laurentiu Mardare, Lidia Benea

**Affiliations:** Competences Center: Interfaces-Tribocorrosion-Electrochemical Systems, Faculty of Engineering, Dunarea de Jos University of Galati, 47 Domneasca Street, 800008 Galati, Romania; laurentiu.mardare@ugal.ro

**Keywords:** particle-reinforcement, corrosion, EH36 naval steel, polymeric coatings, marine corrosion

## Abstract

This research work presents the results obtained from a comparative corrosion evaluation of welded joints on uncoated steel in flat position, welded joints in flat position on steel protected with polymeric film, and welded joints in flat position on steel covered with nanocomposite polymeric film (primer reinforced with TiO_2_ nanoparticles). The electrochemical methods of open circuit potential, linear polarization resistance, and electrochemical impedance spectroscopy were used for corrosion evaluation. The results of the electrochemical tests indicate that titanium oxide reinforcing polymeric film to form nanocomposite layers over naval welded steel increases the corrosion protection of polymeric film as compared with unmodified primer.

## 1. Introduction

Increasing the lifetime and operational safety in the manufacture of marine structures, ships, or onshore and offshore structures requires the achievement of higher quality structures that exhibit better resistance to the action of different degradation phenomena specific to the marine environment [1].

In general, marine and naval structures are made by assembling several steel components. The basic method of joining steel used in ships structures and sea-working structures consists of various welding processes. They offer the advantage of quick jointing, a very good watertightness, high strength, excellent ductility, and good weldability [2,3]. According to Rosado et al., arc welding in the protective gas environment is one of the most used processes in the industry, as it ensures good mechanical and plastic properties of welded joints made of low-alloy steel [4]. Zhao et al. [5] demonstrated that the MAG (metal active gas) process is used to improve the stability of the arc combustion, the appearance of the weld, and the thermal power of the arc. The thermal and physical properties of the different protective gases used in the MAG welding process can influence the metal transfer mode in the joint welded, increasing the CO_2_ concentration and thereby leading to a slight increase in the ferrite content in the welded material due to the chemical composition and the speed of cooling. Pamnani et al. [6] stated in their work that the welding process for each material has a different distribution of the residual effort, depending on the degree of contraction, the cooling rate, and the phase transformation. Ma et al. [7] studied the stress cracking corrosion (SCC) behavior of welded joint E690 in a marine environment using the electrochemical method, and they demonstrated that tensile residual stresses have a negative role as they help to propagate the cracks and contribute to both fatigue failure and stress cracking corrosion. Zhiyu et al. [8] studied the prediction life of welded joints in a marine atmospheric environment with artificial pitting corrosion, showing that with the increasing of artificial pitting corrosion, the fatigue life of welded joints decreased by 50%. Aeran et al. [9] studied a framework to assess the structural integrity of marine structures’ welded joints and concluded that reduction of the lifetime of marine structures’ welded joints is generally caused by the loss in thickness of the metallic material, cross-sectional area, stress concentration effect in the area of corrosion paints, and increased cracking rate. Ahmad et al. [10] studied the corrosion of DH36 (carbon manganese marine steel), and they showed that corrosion affects the performance of marine structures and decreases the resistance of the components. In general, corrosion tests are performed to evaluate the resistance of the material against corrosion in order to estimate the life of the marine structure.

High strength low alloy steel (EH36 HSLA) is used in marine shipbuilding, port installations, onshore constructions, and offshore marine structures in coastal regions due to the good combination of weldability and strength. However, seawater corrosion and the interaction of marine-specific natural forces can cause damage to the structure. Corrosive organic coatings are presented in the literature as a solution for improving the surfaces of metallic materials against corrosion. In the literature, surface improvement by the incorporation of TiO_2_ nanoparticles into the polymeric matrix has been reported by several authors: Benea et al. [11] demonstrated that the addition of TiO_2_ nanoparticles into polymeric matrix increases the corrosion resistance of E32 (low alloyed steel); Deyab et al. [12] showed that TiO_2_ nanoparticles can improve the corrosion and mechanical properties of nanocomposite coatings through particle dispersion; Pour et al. [13] demonstrated that the addition of 1 wt% TiO_2_ in the resin matrix improved the tensile strength and the crack propagation of mild steel; and Wei et al. [14] demonstrate that adding TiO_2_ nanoparticles improve the antimicrobial properties, as well as enhanced hardness and wear resistance. 

A series of studies have been presented recently in the literature regarding the use of epoxy resins in anticorrosive protection: They have a good adhesion to the substrate [15], they provide a barrier effect against aggressive species [16], and they have good chemical stability, good weather resistance, and the ability to maintain high hydrophobicity after immersion in common organic solvents and exposure to UV weather conditions [17,18]. The introduction of ceramic nanoparticles such as TiO_2_ into polymeric resins has a role in increasing corrosion resistance. Corrosion behavior can be improved by incorporating an additional phase compatible with the polymer matrix [19]. Significant improvements in mechanical and thermal properties and corrosion protection have been found [20], as well as a reduced water absorption rate [21], good hydrophobic properties, long-term chemical stability, and good resistance to UV action [22,23,24]. The differences in the quality of the welds generated by the lack of fusion and porosity can have a dynamic effect on the corrosion resistance, which can lead to the failure of the structures made by welded joints. Due to metallurgical and chemical differences and the creation of residual stresses during the welding process (variations in geometry near the welding tip, local stress concentrations), subsequent heating and cooling can change the mechanical properties of the material in this specific area. Moreover, repeated mechanical stresses can reduce the corrosion resistance of welded structural materials.

For these reasons, it is important to improve the corrosion protection coatings to protect the supplement. The principal objective of this research work is to evaluate the effect of dispersed TiO_2_ nanoparticles into polymeric film on the corrosion resistance of (a) unprotected EH36 naval steel, (b) a welded joint on EH36 in flat position (PA), (c) a welded joint in flat position (PA) on EH36 coated with unmodified polymeric primer, and (d) a welded joint in flat position (PA) on EH36 painted with modified polymeric film by the dispersion of TiO_2_ nanoparticles into it. All the surfaces are evaluated to the point of corrosion behavior by immersion into natural seawater. In order to monitor the corrosion process, electrochemical techniques are used. The results show the improvement of anticorrosion protection using a modified primer with dispersed titanium oxide nanoparticles for coated welded joints on EH36 naval steel that is exposed to water from the Black Sea, as compared with unmodified primer or uncoated welded joints.

In situ electrochemical measurements as open circuit potential (OCP), polarization resistance (R_p_), and electrochemical impedance spectroscopy (EIS) are performed to monitor the corrosion process. The results show an enhancement of corrosion resistance for welded joints on EH36 naval steel covered with polymeric layers that have the primer reinforced with TiO_2_ nanoparticles when exposed to the seawater environment as compared with unprotected naval steel and uncoated welded joints.

## 2. Materials and Methods

### 2.1. Materials

**Figure 1:**
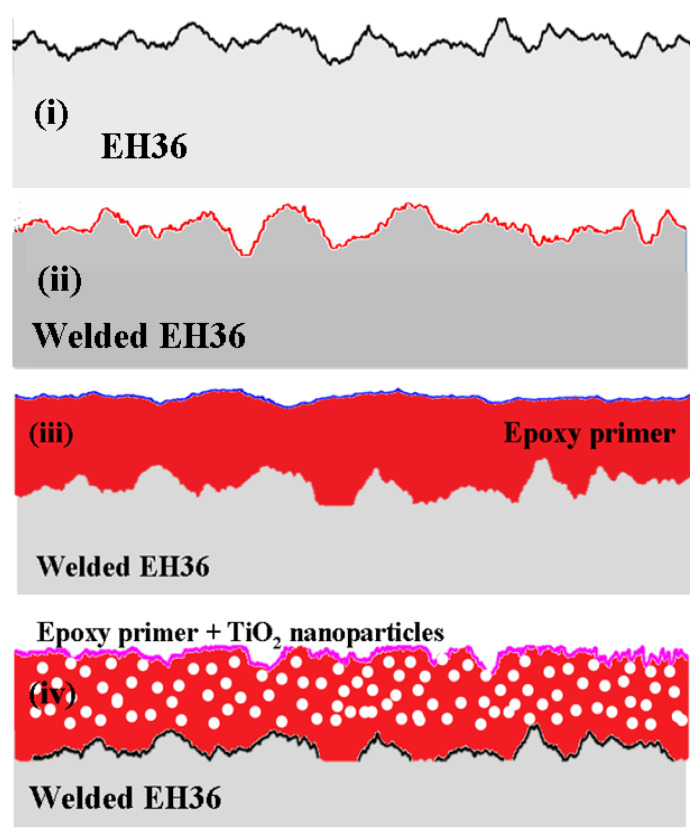
Scheme of studied samples with and without protective layers. (**i**) unprotected EH36 steel; (**ii**) EH36 welded joint without coating; (**iii**) EH36 welded joint coated with epoxy primer; (**iv**) EH36 welded joint covered with polymeric primer with dispersed TiO_2_ nanoparticles.

Four types of samples with EH36 nonprotected steel for shipbuilding were used (Figure 1): (i) EH36 nonprotective steel used as the base material with the code “EH36PM”; (ii) EH36 welded head joint in the PA/1G (welding in flat position/flat groove weld) position without protective cover with sample code “EH36PAWJ” (Arcelor Mittal, Galati, Romania); (iii) EH36 welded joint in the PA/1G position covered with epoxy primer (primer), code “EH36PAWJEP” (Arcelor Mittal, Galati, Romania); and (iv) EH36 welded head joint in the PA/1G position covered with epoxy paint in which TiO_2_ nanoparticles were dispersed, code “EH36PAWJEP + TiO_2_” (Arcelor Mittal, Galati, Romania). The thickness of the applied organic coatings was measured with a PCE-CT 28 (PCE Instruments Co., Southampton, UK) gauge, and we made dry films with a constant thickness of 100 µm (Table 1).

### 2.2. Preparation of Welded Samples

To make welded joints, EH36 steel material was used, whose chemical composition is found in Table 2. In Table 3, the mechanical properties of EH36 steel are shown.

EH36 steel was used as the base material to achieve a welded joint in PA position. The PA/1G is a flat position, in which the welder has the piece right below the torch.

For welding, the following were used: EH36 steel sheets of 10 mm thick, tubular steel wires with E70C-6MH4 (Lincon Electric Ductil, Buzau, Romania), low fume metal powder core tubular steel E81T1-Ni1MJH4 (Lincon Electric Ductil, Buzau, Romania) rutile flux-cored wires with a 1.2 mm diameter, and Corgon 18 (is a two-component shielding gas mixture comprising 82% argon and 18% carbon dioxide) (Lincon Electric Ductil, Buzau, Romania), which is a two-component shielding gas mixture comprising 82% argon and 18% carbon dioxide, as an auxiliary material. The chemical compositions of the filler materials can be found in Table 4.

**Figure 2:**
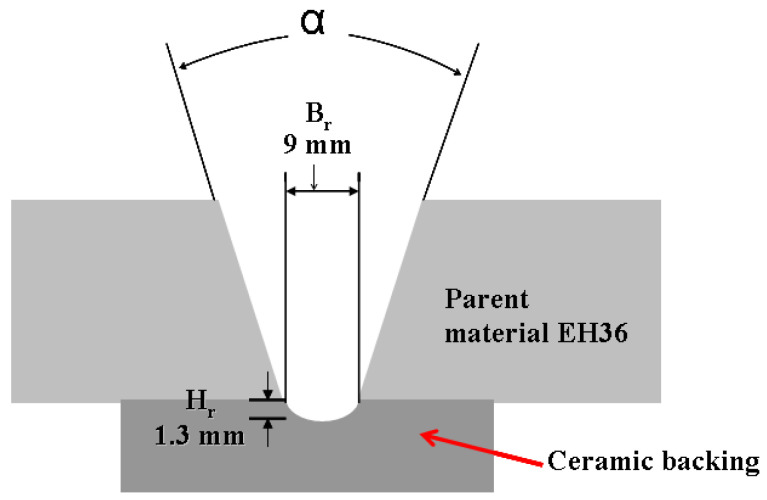
Preparation of components for welding.

Samples welded to the PA/1G welding position for a 10 mm welded joint were made on a flat ceramic support with concave channel (width of ceramic support channel) = 9 mm and Hr (ceramic support groove depth) = 1.3 mm) and with the joint opening of the welding components, with a narrow V-shaped joint (b = 5 mm and α ±= 40°) according to Figure 2. To make the samples, the Phoenix 405 Progress pulse MM TDM welding equipment (EWM Group, Göttingen, Germany) and the K-BUG 5102 welding machine (Weld Tooling Corporation, Canonsburg, USA) were used.

### 2.3. Fabrication of Modified Epoxy Primer Reinforced with TiO_2_ Dispersed Nanoparticles

**Figure 3:**
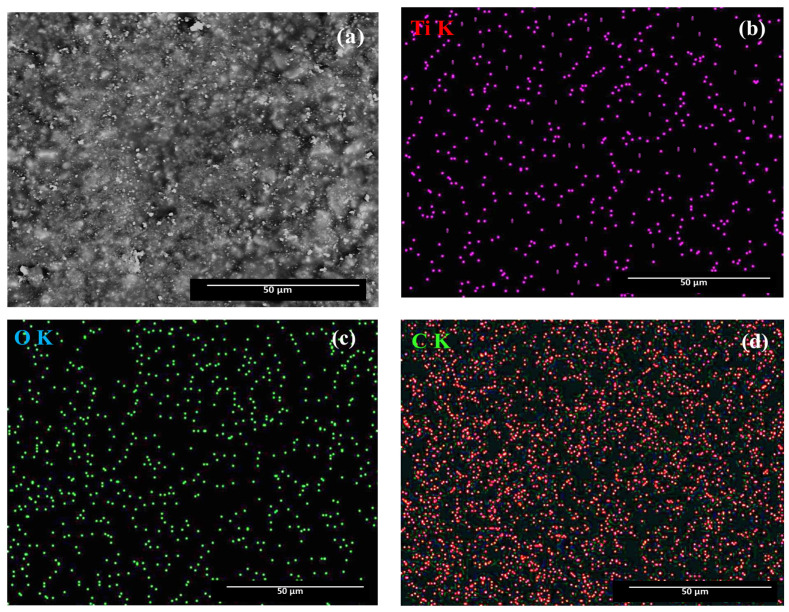
(**a**) SEM image of TiO_2_ dispersed nanoparticle in the epoxy primer; EDX mapping and elemental analysis, including (**b**) titanium, (**c**) oxygen, and (**d**) carbon elemental analysis.

The epoxy primer (Intergard 269) without and with TiO_2_ (purchased from Sigma-Aldrich, St. Louis, MO, USA, 99.5 wt%) dispersed nanoparticles was obtained in cylindrical chemical cell at room temperature, under ultrasonic stirring (UP100H, Hielscher, Teltow, Germany) in order to maintain a homogenous dispersion. Epoxy resin (purchased from Ciba Co, Basel, Switzerland) and the hardening agent (purchased from Ciba Co) were mixed for 50 min in a 5:1 mass ratio. The average diameter of the TiO_2_ nanoparticles used was approximately 100 nm, and these nanoparticles were first dispersed in an organic solvent (GTA 220 benzyl alcohol purchased from International Paint, Constanta, Romania, p.a. purity). The dispersion was magnetically stirred for 50 min and then added into a mixture of epoxy resin and hardening agent at a concentration of 0.5% weight. A SEM-EDX (Scanning electron microscopy-Energy-dispersive X-ray spectroscopy) (model FEI QUANTA 200, FEI Company, Eindhoven, Holland) characterization on the quality of TiO_2_ nanoparticles dispersed in the epoxy primer is presented in the Figure 3.

The elemental mapping results indicate the existence of elements Ti, O, and C, suggesting that TiO_2_ nanoparticles were uniformly dispersed into the epoxy primer. A uniform distribution of Ti atoms could be observed throughout the entire reinforced polymeric surface.

**Figure 4:**
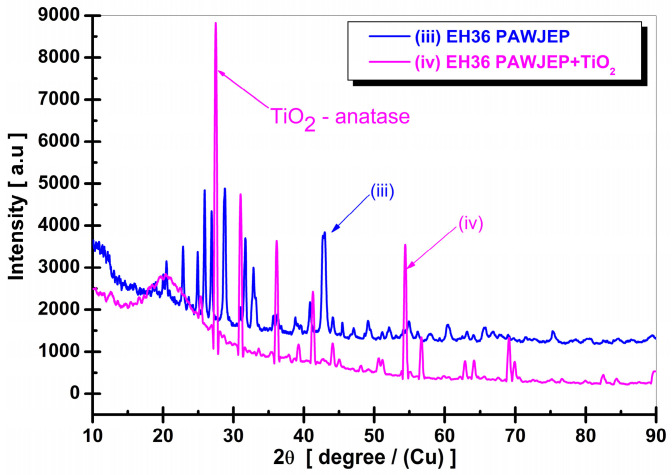
XRD patterns of the coatings system: (iii) polymeric primer; (iv) polymeric primer reinforced with TiO_2_ nanoparticles.

In Figure 4, the XRD patterns of the epoxy primer (Sample 3) and epoxy primer reinforced with TiO_2_ nanoparticles are presented. The epoxy primer pattern was shifted with a 1000 a.u. intensity in order to be better observed. The main basic compound of the epoxy primer is 2-[2-(hydroxymethyl)phenyl]-1-phenylethanol (C_15_H_16_O_2_), which appears at many instances of the 2θ degree. Other organic compounds also appear, such as C_4_H_10_O, C_8_H_10_, C_6_H_12_O_6_, and BaSO_4_ as primer pigment.

The principal peak corresponding to TiO_2_ nanoparticles as anatase was identified with the help of program Match! Version 3 (http://www.crystalimpact.com/match (accessed on 16 January 2021), Bonn, Germany) and Dron 3 equipment (BV Alrosa Group, Sankt-Petersburg, Russia), with the corresponding angle at 2θ = 26.40°. This detected phase was recorded in the database of the program Crystallography Open Database (COD, http://crystallography.net (accessed on 16 January 2021)) 96-101-0943, belonging to the tetragonal crystallization system, space group I41/amd, on the XRD patterns corresponding to the polymeric primer reinforced with TiO_2_ nanoparticles (Sample 4).

### 2.4. Roughness and Microhardness of the Studied Samples

The surface roughness profile of the samples resulted from the surface measurements performed with a Mitutoyo Surftest SJ-210 (Mitutoyo Corporation, Kanagawa, Japan) surface roughness tester. The microhardness of all types of surface was measured using a PMT-3 gauge (Optomechanical Corp., Saint-Petersburg, Russia) by the Vickers method. Vickers hardness values were determined using an indenter of 0.1 kgf/mm^2^.

### 2.5. Electrochemical Measurements for Corrosion Investigations

The corrosion investigations were performed in natural sea water from the Black Sea at a 5 m depth. The sampling geographical coordinates of the Black Sea location were N 44050′30.6809″ and E 29045′31.0606″. The main seawater parameters are listed in Table 5.

**Figure 5:**
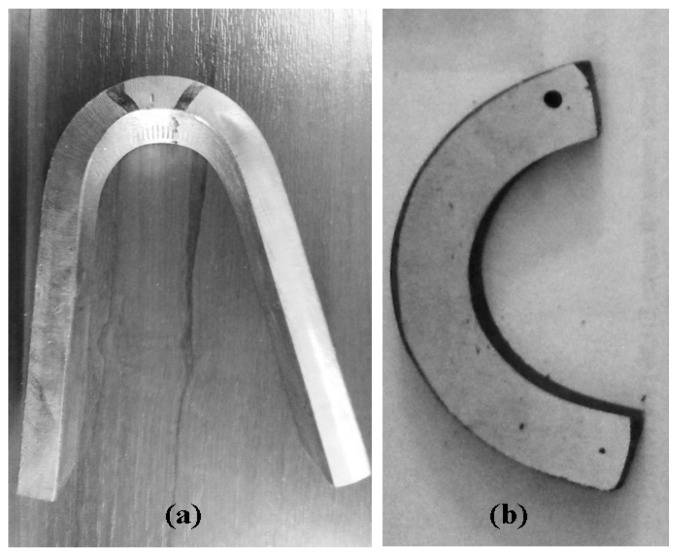
Tested samples: (**a**) sample EH36PAWJ after cross-bending with compressed root, (**b**) EH36PAWJ after sampling and surface grinding.

Here, a PSU unit denotes a practical salinity unit, which is the salt content expressed as grams of salt per 1 kg seawater (g salt/kg seawater). Each sample for the corrosion test was a test specimen with a defined surface area, with other surfaces being isolated using a nonconductive silicone resin. The welded joint samples, shown in Figure 5, were taken after the mechanical test was performed. After sampling, one of the front surfaces of the specimens was rectified.

All uncovered samples were degreased with organic solvents. Samples covered with the epoxy primer were degreased with detergent dissolved in water.

For the corrosion resistance evaluation, we used a standard corrosion cell as the working electrode (the tested samples), while a Pt–Rh grid used as an auxiliary electrode and an Ag/AgCl electrode (saturated KCl solution, E (potential) = 199 mV vs. normal hydrogen electrode (NHE)) was taken as a reference electrode. The open circuit potential was monitored for each sample during immersion time. The linear polarization curves were measured for 50 times in order to have the evolution of polarization resistance during immersion time. The polarization resistance evaluation was performed by applying a linear polarization method around the free potential value with very low potential difference (±40 mV) to maintain a stationary state surface.

Electrochemical impedance spectroscopy was measured on an electrochemical workstation PGZ 100 (Radiometer Analytical SAS, Villeurbanne, France). The experimental data (EIS) were obtained at the open circuit potential in the frequency range of 100 kHz to 10 mHz, with an amplitude of the sinusoidal signal of 10 mV. The experimental data that EIS obtained were fitted with equivalent electrical circuits depicted in Figure 4 for the evaluation of the polarization resistances of each studied surface.

**Figure 6:**
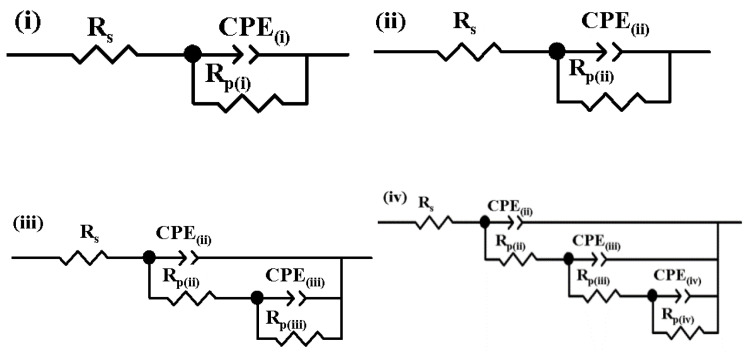
Equivalent circuits used to simulate and model the resulted data of the electrochemical impedance spectroscopy (EIS) spectrum obtained in seawater for (**i**) unprotected EH36 steel; (**ii**) EH36 welded joint without coating; (**iii**) EH36 welded joint coated with epoxy primer; (**iv**) EH36 welded joint covered with polymeric primer with dispersed TiO_2_ nanoparticles.

The equivalent circuit model depicted in Figure 6i,ii is a single time constant circuit used to describe the electrochemical behavior of the EH36PM and EH36PAWJ sample surfaces, respectively, as uncovered by simulated EIS data. The elements R_s_, R_p(i)_, and R_p(ii)_ describe the ohmic resistance of the electrolyte and the polarization resistances, respectively, of the uncoated steel and welded steel joints. The constant phase elements (CPEs), i.e., CPE_(i)_ and CPE_(ii)_, are the phase elements that take into account the nonideal capacitive behavior of the studied surfaces, which in the case of uncovered steel and welded steel can be attributed to the surface heterogeneity due to surface roughness.

A good match is seen between the experimental data and those obtained via simulation by parallel CPE and R_p_, which were arranged to depict the charge transfer process at the uncovered steel/seawater interface of the EH36PM sample. The experimental and the simulated data were highlighted by obtaining the values of the matching factor chi-square (χ^2^), which was less than 10^−3^. The corrosion behavior of the primer surface EH36PAWJEP was simulated with the equivalent circuit shown in Figure 6iii. This circuit depicts two electrochemical interfaces that are shown in the primer covered steel EH36PAWJEP. A pair of CPE and R elements was used in parallel to replace the dielectric properties of the epoxy primer film, namely the capacitance, CPE_(iii)_, and the polarization resistance, R_p(iii)_. The electrochemical corrosion behavior of the steel covered EH36PAWJEP + TiO_2_ sample, whose surface was coated with a nanocomposite epoxy primer with the TiO_2_ dispersed nanoparticles, was simulated with an equivalent circuit shown in Figure 6iv.

The pair of CPE_(iv)_ and R_p(iv)_ elements was used in parallel to match the insulation properties of the epoxy primer composite film with TiO_2_ dispersed particles, i.e., the capacitance and polarization resistance, respectively. The ceramic TiO_2_ nanoparticles demonstrated that they can have an insulating effect in the polymeric matrix where they are dispersed into. The circuit elements are defined in Figure 6 as follows: R_s_ is the resistance of the solution, R_p_ represents the polarization resistance (related to the electron transfer), and CPE represents the constant phase element corresponding to the capacitance of each surface type. The experimental data were acquired with specific software, i.e., VoltaMaster 4 (Radiometer Analytical SAS, Villeurbanne, France). For checking reproducibility, each experiment was repeated at least three times. The modeling, simulation, and fitting of the experimental data of electrochemical impedance spectroscopy data to obtain the electrical equivalent circuits of the studied surfaces were performed with the Zview software (Scribner Associates, Lyon, France).

## 3. Results and Discussion

### 3.1. Roughness and Microhardness of the Surfaces Tested

The results of the roughness and microhardness evaluations are listed in Table 6 for each surface type. The arithmetical mean (R_a_) values of surface roughness were in the range of 2.485–3.125 µm. However, for the EH36PAWJEP + TiO_2_ sample coated with TiO_2_ dispersion modified primer, the average value of surface roughness was much smaller, i.e., R_a_ = 1.753 µm, confirming a development in the surface quality from this point of view. The Vickers hardness values for the EH36PM and EH36PAWJ samples determined using an indenter of 0.1 kgf/mm^2^ were with an order of magnitude higher than those of the EH36PAWJEP and EH36PAWJEP + TiO_2_ samples. This means a lower mechanical resistance of the primer coated samples.

### 3.2. Electrochemical Measurements

#### 3.2.1. Time Evolution of Open-Circuit Potential (OCP)

**Figure 7:**
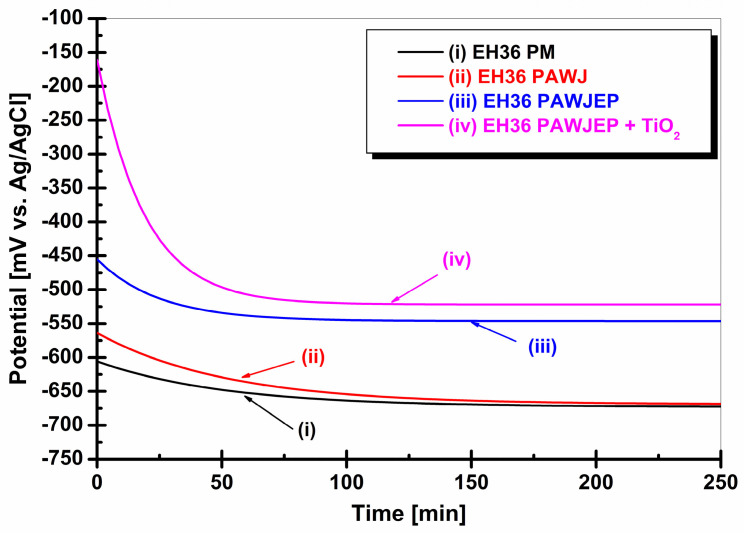
Evolution of open-circuit potential versus immersion time in seawater for: (i) EH36PM without protective coating used like parent material, (ii) EH36PAWJ without protective coating, (iii) EH36PAWJEP coated with polymeric primer and (iv) EH36PAWJEP + TiO_2_ coated with nanocomposite polymeric primer with dispersed TiO_2_ nanoparticles.

Corrosion tests were initiated by monitoring the free potential evolution for 250 min for each sample during immersion into seawater until a constant state value was reached, as presented in Figure 7.

For the EH36PM naval steel, the OCP trend was slightly shifted to more negative values than in the initial immersion, i.e., from E = −640 mV to −685 mV vs. Ag/AgCl at the end. The free potential shifts to the negative direction indicate that this material is not capable of forming an oxide protective layer on its surface after exposure to the seawater environment, being corroded down by dissolving.

For the EH36PAWJ welded joint without protection coating, we found the same trend of smooth displacement of the OCP in the negative direction from the initial immersion value of E = −563 mV vs. Ag/AgCl at a value of −668 mV vs. Ag/AgCl, with equilibrium being reached after 210 min. A slight shift of the potential towards the negative direction was also seen for the welded joint EH36PAWJEP covered with epoxy primer, with the potential starting from an initial value of E = −455 mV vs. Ag/AgCl. The steady state was reached after 150 min, and a value of −546 mV vs. Ag/AgCl was recorded.

For the surface of the epoxy coated EH36 welded joint where TiO_2_ nanoparticles, EH36PAWJEP + TiO_2_, were added, the free potential value ranged from E = −160 mV to E = −521 mV as compared to Ag/AgCl. The free potential of this surface reached the stationary state after 80 min, indicating the formation of a protective film. The free potential of this surface in an uncoated state has no noble potential and therefore less corrosion resistance to the aggressive marine environment.

In the case of the welded EH36PAWJ joint, the steady state of the free potential was reached at the same value as uncoated steel. The two types of polymeric protective layers applied to the EH36PAWJEP welded joints covered with epoxy primer and the EH36PAWJEP + TiO_2_ covered with epoxy primer, in which TiO_2_ dispersed nanoparticles were added, provide an improvement in the corrosion resistance performance.

#### 3.2.2. Polarization Resistance (R_p_) Determined by Linear Polarization Curve

The polarization resistance is the only corrosion monitoring method that allows for the measurement of corrosion rates directly in real time. Thus, a number of 50 polarization curves, which consisted of 50 values of polarization resistance and corrosion rates respectively, were measured by applying the Stern–Geary equations and the Tafel slopes to each recorded linear polarization curve [25]. The corrosion currents estimated using these methods can be converted into corrosion rates expressed as penetration rates using Faraday’s law.

**Figure 8:**
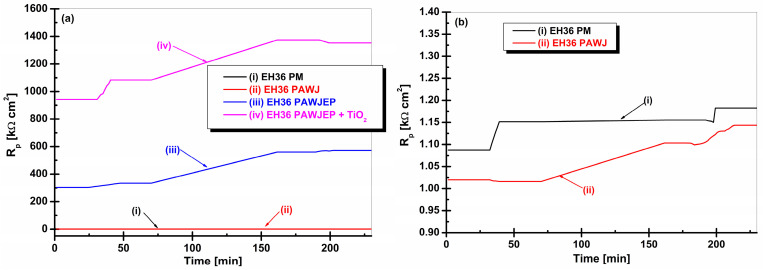
The evolution of R_p_ values during immersion time (**a**) of: (i) EH36PM without protective coating used like parent material, (ii) EH36PAWJ welded steel without protective coating, (iii) EH36PAWJEP coated with polymeric primer and (iv) EH36PAWJEP + TiO_2_ coated with nanocomposite polymeric primer reinforced with dispersed TiO_2_ nanoparticles and (**b**) zoom in low polarization resistance value for (i) and (ii).

From Figure 8, it can be seen that the minimum value of the polarization resistance (R_p_) was reached by the naval steel EH36PM being equal to 1.044 kΩ cm^2^. This R_p_ value for the EH36PM naval steel remained unchanged over the seawater immersion period. The average polarization resistance value for EH36PAWJ was 1.039 kΩ cm^2^. For EH36PAWJEP covered with epoxy primer, the mean value of R_p_ was 572.15 kΩ cm^2^. EH36PAWJEP + TiO_2_ covered with epoxy primer (in which TiO_2_ dispersed nanoparticles were added) achieved the highest polarization resistance value of 1352.80 kΩ cm^2^.

Since the curves of the polarization resistance evolution for EH36PM and EH36PAWJ have close values and are not clearly observed in Figure 8a, they are shown separately in Figure 8b as a zoom-in copy of Figure 8a in the low values of polarization resistances.

**Figure 9:**
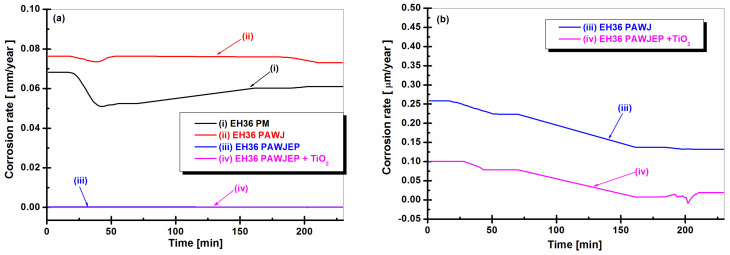
Evolution of corrosion rate during immersion period (**a**) for: (i) EH36PM without protective coating used like parent material, (ii) EH36PAWJ welded steel without protective coating, (iii) EH36PAWJEP coated with polymeric primer and (iv) EH36PAWJEP + TiO_2_ coated with nanocomposite polymeric primer reinforced with dispersed TiO_2_ nanoparticles and (**b**) in low corrosion rate value for (iii) and (iv).

The corrosion rate expressed as the penetration index is inversely proportional to the polarization resistance and may be directly calculated from R_p_ by using the density of the metallic material. The evolution in time of this form of corrosion rate is shown in Figure 9a,b.

From the data depicted in Figure 9, it can be observed that the highest corrosion rate corresponds to EH36PAWJ when compared to the other analyzed samples. Corrosion rates recorded and expressed in μm/year were 70 μm/year for EH36PM, 0.76 μm/year for EH36PAWJ, a mean value of 0.13 μm/year for EH36PAWJ coated with epoxy primer, and a mean value of 0.018 μm/year for EH36PAWJ coated with epoxy primer reinforced with TiO_2_.

#### 3.2.3. Electrochemical Impedance Spectroscopy (EIS)

Electrochemical impedance spectroscopy (EIS) is the most efficient method used for obtaining substantial information on the corrosion protection mechanism provided by the protective films formed on the surface of materials.

**Figure 10:**
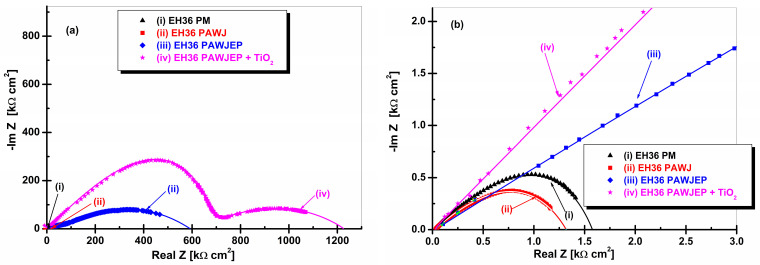
Electrochemical impedance spectroscopy diagrams in Nyquist representation for investigated samples in seawater on large frequency range (**a**) and zoom in low frequency range (**b**) for: (i) EH36PM without protective coating used like parent material, (ii) EH36PAWJ without protective coating, (iii) EH36PAWJEP coated with polymeric primer and (iv) EH36PAWJEP + TiO_2_ coated with nanocomposite polymeric primer with dispersed TiO_2_ nanoparticles. The plain symbols represent the experimental data, while the lines represent the fitting diagrams.

Figure 10a shows the highest impedance values obtained when steel is covered with primer mixed with TiO_2_ dispersed nanoparticles (code EH36PAWJEP + TiO_2_). The steel coated with primer, EH36PAWJEP, offers a lower polarization resistance (half circle diameter) compared to that of the primer mixed with TiO_2_ dispersed nanoparticles.

**Figure 11:**
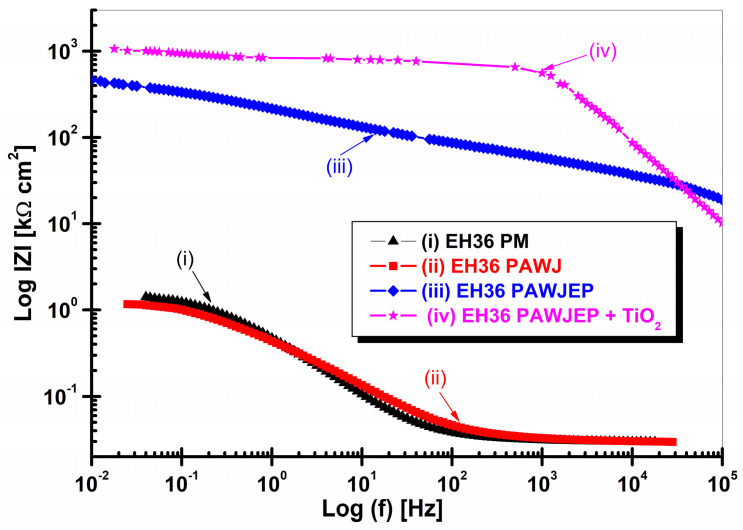
Electrochemical impedance spectroscopy diagrams in Bode representation as impedance modulus Z vs. log frequency for: (i) EH36PM without protective coating used like parent material, (ii) EH36PAWJ without protective coating, (iii) EH36PAWJEP coated with polymeric primer and (iv) EH36PAWJEP + TiO_2_ coated with nanocomposite polymeric primer with dispersed TiO_2_ nanoparticles.

**Figure 12:**
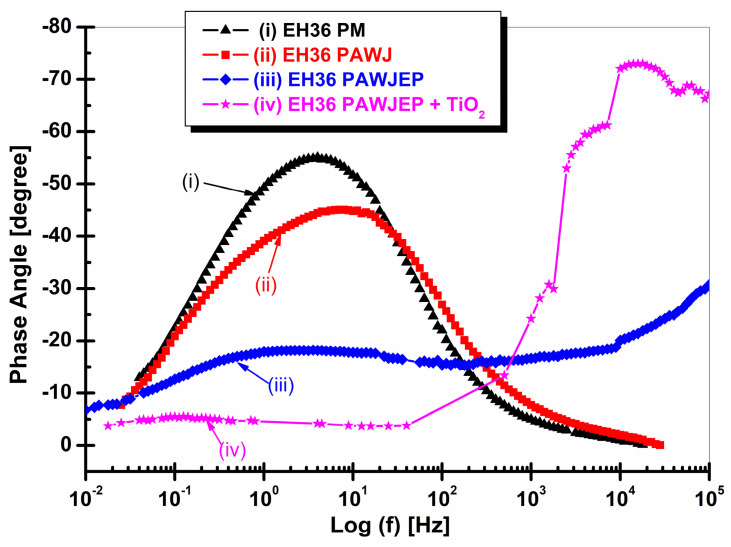
Electrochemical impedance spectroscopy diagrams in Bode representation as Phase angle vs. log frequency for: (i) EH36PM without protective coating used like parent material, (ii) EH36PAWJ welded steel without protective coating, (iii) EH36PAWJEP coated with polymeric primer and (iv) EH36PAWJEP + TiO_2_ coated with nanocomposite polymeric primer with dispersed TiO_2_ nanoparticles.

The EIS data were well established in both Nyquist and Bode representations as the impedance module and the phase angle in relation with the frequency logarithm, as shown in Figure 11 and Figure 12.

The values corresponding to the equivalent electrical circuit proposed in Figure 4 for the simulation of the experimental data (EIS) are presented in Table 7. From the EIS diagrams presented, it can be concluded that TiO_2_ nanoparticles have a significant role in increasing the corrosion resistance of the epoxy primer, namely in displaying the highest corrosion resistance, which highlights the high degree of protection of the nanocomposite layer.

It can be seen that the EIS results are consistent with OCP vs. time, as well as with the polarization resistance values resulted from linear polarization measurements, from which the evolution of polarization resistance over time was extracted.

## 4. Conclusions

Monitoring the free potential in seawater for the four surfaces, i.e., steel without protective coating, a welded joint uncovered, a welded joint covered with polymeric (epoxy) primer, and a welded joint covered with polymeric primer in which dispersed TiO_2_ nanoparticles, has shown a shift in the free potential to more noble values up to the composite coating. The addition of TiO_2_ nanoparticles to a commercial epoxy primer increased the corrosion protection of welded steel, as shown by the change of polarization resistance.

The EIS results indicate that TiO_2_ nanoparticles added in the epoxy primer to form nanocomposite coatings enhanced the corrosion protection performance of the polymeric primer relative to the epoxy-unmodified primer. Due to the insulating effect of the TiO_2_ nanoparticles dispersed into the polymeric primer, the transport routes for the seawater electrolyte to pass through the applied coating system were reduced. In addition, the EIS results are consistent with those obtained by the linear polarization method to calculate the polarization resistance during immersion time. The addition of a low concentration of TiO_2_ nanoparticles significantly improves the corrosion resistance of standard primer coatings.

Polymeric primer coatings reinforced with TiO_2_ nanoparticles revealed an enhanced corrosion resistance when they were applied on EH36 welded steel exposed to a marine corrosive environment. Thus, it was proven that TiO_2_ dispersed nanoparticles have a significant contribution in the corrosion protection of the modified primer layer.

## Figures and Tables

**Table 1:** Samples used for corrosion resistance evaluation.

Sample Number	Sample Code	Surface State	Coating Thickness
i	EH36PM	Unprotected EH36 steel (Figure 1i)	-
ii	EH36PAWJ	EH36 welded joint without coating (Figure 1ii)	-
iii	EH36PAWJEP	EH36 welded joint coated with epoxy primer, (Figure 1iii)	100 μm
iv	EH36PAWJEP + TiO_2_	EH36 welded joint covered with polymeric primer with dispersed TiO_2_ nanoparticles (Figure 1iv)	100 μm

**Table 2:** Chemical composition of EH36 HSLA steel.

Material	C	Mn	Si	P	S	Al	Nb	V	Ti	Cu	Cr	Ni	Mo
EH36	0.06	0.38	0.25	0.01	0.004	0.024	0.04	0.05	0.02	0.02	0.02	0.02	0.01

**Table 3:** Mechanical characteristics of HSLA EH36 steel.

Yielding Point, R_p0,2_	Tensile Strength, R_m_ [MPa]	Breaking Elongation, A_5_ [%]
426	550	27

**Table 4:** Chemical composition of welding wire.

Type/Composition	C	Mn	Si	P	S	Al	Nb	V	Cu	Cr	Ni	Mo
ER70S-6	0.06	1.40	0.80	0.02	0.035	0.008	0.10	0.03	0.50	0.15	0.15	0.15
E81T1Ni1MJH4	0.06	1.39	0.53	0.009	0.008	0.006	0.009	0.01	0.18	0.02	1.00	0.03

**Table 5:** Seawater characteristics.

Salinity [PSU]	Electrical Conductivity [S/m]	pH
15.2983	2.22	8.43

**Table 6:** Values of surface roughness and Vickers microhardness, HV_0.1_.

Sample code	EH36PM	EH36PAWJ	EH36PAWJEP	EH36PAWJEP + TiO_2_
HV_0.1_	167	211	21.1	22.4
R_a_ [µm]	2.864	3.125	2.485	1.753

**Table 7:** The equivalent circuit elements values used for simulation of experimental data.

Elements of Equivalent Circuit	EH36PM	EH36PAWJ	EH36PAWJEP	EH36PAWJEP + TiO_2_
R_s_ [Ω cm^2^]	29.75			
CPE_(i)_ [F/cm^2^]	0.00047193			
α_(i)_	0.75			
R_p(i)_ [Ω cm^2^]	1575			
χ^2^	10^−3^			
R_s_ [Ω cm^2^]		28.76		
CPE_(ii)_ [F/cm^2^]		0.00057763		
α_(ii)_		0.65		
R_p(ii)_ [Ω cm^2^]		1293		
χ^2^		10^−3^		
R_s_ [Ω cm^2^]			29.87	
CPE_(ii)_ [F/cm^2^]			1.4167 × 10^−7^	
α_(ii)_			0.41	
R_p(ii)_ [Ω cm^2^]			67,000	
CPE_(iii)_ [F/cm^2^]			2.1571 × 10^−6^	
α_(iii)_			0.37	
R_p(iii)_ [Ω cm^2^]			525,000	
χ^2^			10^−3^	
R_s_ [Ω cm^2^]				28.49
CPE_(ii)_ [F/cm^2^]				5.416 × 10^−7^
α_(ii)_				0.89
R_p(ii)_ [Ω cm^2^]				1170,000
CPE_(iii)_ [F/cm^2^]				1.4641 × 10^−6^
α_(iii)_				0.75
R_p(iii)_ [Ω cm^2^]				41,000
CPE_(vi)_ [F/cm^2^]				4.3766 × 10^−6^
α_(vi)_				0.35
R_p(vi)_ [Ω cm^2^]				49,000
χ^2^				10^−3^

χ^2^—chi-square, α—capacitance.

## Data Availability

The data presented in this study are available on request from the corresponding author.
